# Not Just a Cyst: A Draining Gluteal Lesion Revealing a Rectocutaneous Fistula in Ulcerative Colitis

**DOI:** 10.14309/crj.0000000000002179

**Published:** 2026-06-10

**Authors:** Ryan Sasse, Noor Hassan, Maaz Hassan, Valerica Mateescu, Tahar Malek Mahmoudi, Esmat Sadeddin

**Affiliations:** 1 University of Missouri-Kansas City School of Medicine, Kansas City, MO; 2 Department of Gastroenterology, University of Missouri-Kansas City, Kansas City, MO; 3 Department of Radiology, University of Kansas Medical Center, Kansas City, KS; 4 Department of Pathology, University of Missouri-Kansas City, Kansas City, MO

**Keywords:** ulcerative colitis, rectocutaneous fistula, inflammatory bowel disease, perianal disease, extraintestinal manifestation

## Abstract

We report a 48-year-old man with poorly controlled ulcerative colitis (UC) who presented with severe rectal pain, hematochezia, and a chronically draining lesion on the left buttock. Initial cross-sectional imaging revealed active colitis, and targeted ultrasound of the left buttock suggested a benign epidermal inclusion cyst. Colonoscopy demonstrated continuous Mayo 3 proctosigmoiditis with a normal proximal colon and terminal ileum. While he clinically improved with intravenous corticosteroids, the gluteal lesion persisted. Before discharge, pelvic magnetic resonance imaging unexpectedly revealed a rectocutaneous fistula extending from the distal rectum into the left gluteal soft tissues. This case illustrates that, although rare, rectocutaneous fistulas can complicate UC. Clinicians should include fistulizing disease in the differential diagnosis of any chronically draining perianal or gluteal lesion in UC, and consider early pelvic magnetic resonance imaging when symptoms are discordant with endoscopic findings or fail to resolve with standard therapy.

## INTRODUCTION

Perianal disease (PAD) is a well-recognized complication of Crohn's disease, with perianal fistulas affecting roughly 20%–25% of patients over the disease course.^1^ This burden is secondary to the nature of Crohn's disease, where transmural inflammation promotes deep fissuring ulcers and penetrating sinus tracts.^2^ Outside this setting, most perianal fistulas are cryptoglandular in origin, arising from infection and obstruction of the anal glands with subsequent anorectal abscess formation and chronic fistulous tract development.^3–5^ Less common causes include obstetric trauma or iatrogenic injury resulting in rectovaginal or anovaginal fistulas, while other contributors include previous pelvic or anorectal surgery, malignancy, radiation injury, hidradenitis suppurativa, or atypical infections.^6–9^

By contrast, PAD is much less common in ulcerative colitis (UC), with cumulative incidence estimates ranging from 5 to 16% over 5-10 years.^10,11^ Among affected patients, only 0.9-3.4% develop fistulizing disease.^11,12^ This relative rarity reflects the underlying pathophysiology of UC, in which inflammation is typically confined to the mucosa and submucosa. When fistulas do occur, it is thought to arise in the setting of severe or longstanding inflammation, with deep ulceration, tissue destruction, and secondary extension. Here, we present a case of severe left-sided UC in which a chronically draining buttock lesion was ultimately recognized as a rectocutaneous fistula on the final hospital day. This case highlights the need to maintain fistulizing disease in the differential diagnosis of any persistent, draining, nonhealing perianal or gluteal lesion in patients with UC.

## CASE REPORT

A 48-year-old man with a 7-year history of UC presented as an interfacility transfer for evaluation of severe rectal pain, hematochezia, and a chronically draining lesion on the left buttock. Approximately 4 months prior, he experienced an episode of rectal pain that resolved spontaneously, followed by the appearance of this lesion. He was originally diagnosed with UC at an out-of-state hospital with endoscopic and histologic confirmation. Per his report, he achieved clinical remission with a course of systemic corticosteroids, although outside records detailing the initial extent and severity of disease were unavailable. Since that time, he remained clinically quiescent on maintenance sulfasalazine 500 mg twice daily as his only UC-directed therapy, without documented flares, hospitalizations, treatment escalation or prior PAD. In the months preceding this presentation, he reported taking sulfasalazine only 1 to 2 times per week. He denied nonsteroidal anti-inflammatory drug use, tobacco use, or alcohol misuse, with no history of obesity (body mass index 22 kg/m^2^) or diabetes. His dermatologic and infectious history was notable for a left medial malleolar soft tissue infection requiring antibiotics 3 years ago, and a right-hand deep space abscess requiring 4 operative incision-and-drainage procedures 2 years ago; he has no other surgical history to suggest additional risk factors of PAD.

By the time of this admission, the left gluteal lesion had been recurring over the preceding months, intermittently enlarging and draining serosanguinous fluid. He reported a short outpatient course of oral antibiotics but was unable to recall the specific agents; these provided only subjective, transient improvement, and the lesion never fully resolved. He did not perceive that the size or tenderness of the lesion worsened in parallel with changes in his bowel symptoms, instead stating that it would simply fill up and then drain intermittently. On arrival, he was afebrile and hemodynamically stable. Examination was notable for discomfort with sitting due to rectal pain with an otherwise unremarkable abdominal examination. Inspection of the left gluteal/perianal region revealed a firm, indurated nodule in the left posterior perianal quadrant, a few cm from the anal canal (Figure 1). There was no obvious fluctuance or surrounding cellulitis.

**Figure 1.**
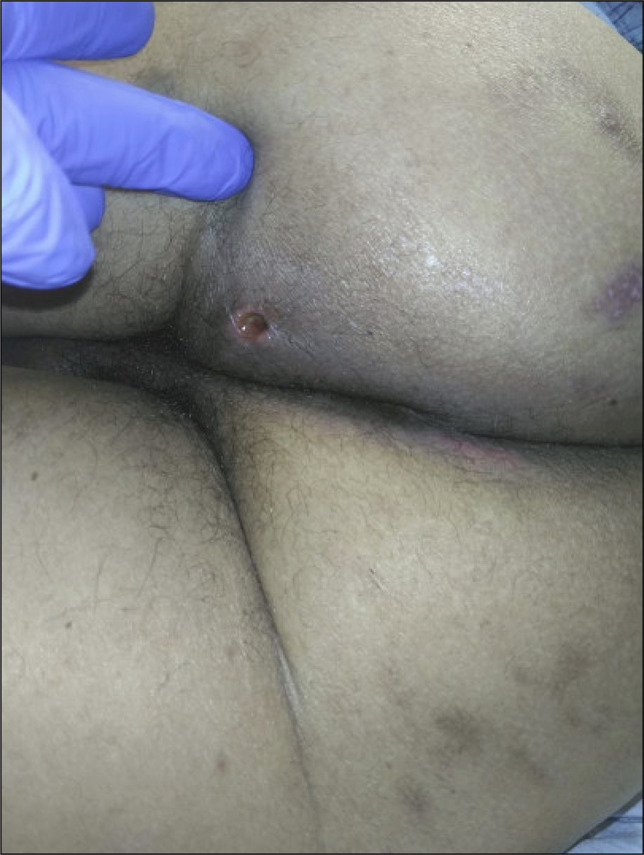
External photograph of the left gluteal/perianal region showing the draining cutaneous opening corresponding to the distal end of the fistula tract.

At the referring hospital, abdominal/pelvic computed tomography demonstrated rectal, sigmoid, and distal descending colitis consistent with UC, without a discrete abscess. A targeted soft-tissue ultrasound of the left buttock showed a 1.6 cm hypoechoic lesion with peripheral vascularity and an internal hyperechoic focus, interpreted as a likely benign epidermal inclusion cyst, with possible superimposed infection, but no clear fistulous tract. The original computed tomography and ultrasound images from the referring facility were not available for independent review at our institution, and our assessment therefore relied on the formal radiology reports.

Initial laboratory tests showed a hemoglobin of 10.7 g/dL, thrombocytosis, and a markedly elevated C-reactive protein of 131.6 mg/L. Stool pathogen polymerase chain reaction and *Clostridioides difficile* assays were negative. He was started on intravenous methylprednisolone 40 mg daily for a presumed severe UC flare. Esophagogastroduodenoscopy revealed erythematous gastritis and mild duodenitis with nonbleeding erosions. Colonoscopy demonstrated continuous, severe ulcerative proctosigmoiditis from the anal verge to 35 cm, with erythema, edema, granularity, spontaneous bleeding, and deep linear ulcerations (Mayo 3), while the remaining colon and terminal ileum were normal. Rectal retroflexion revealed only hemorrhoidal skin tags and no obvious perianal fistula. Colonoscopic images and representative histology are shown in Figure 2.

**Figure 2.**
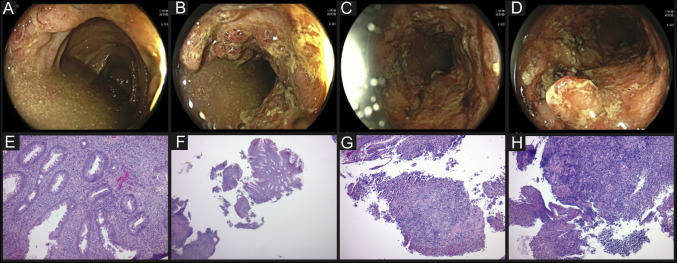
Colonoscopic images demonstrating continuous severe proctosigmoiditis (Mayo 3) from the anal verge to approximately 35 cm. Panels (A–D) correspond to progressively more distal views, with diffuse erythema, friability, fibrinous exudate, and deep linear ulcerations. Panel (E): Sigmoid colon biopsy with marked active chronic colitis (H&E, 400×). Panels (F–H) Rectal biopsy with marked active chronic proctitis with cryptitis, crypt abscesses, ulceration, fibrinopurulent exudate, and granulation tissue (H&E, F: 100×, G and H: 400×). The proximal colon and terminal ileum were endoscopically normal (not shown). H&E, hematoxylin and eosin.

Over the next several days, his bowel frequency decreased with diminishing hematochezia, and C-reactive protein trended downward from 131.6 to 50.5 and then 20.6 mg/L. Despite improvement in luminal disease, he continued to express concern regarding this left buttock lesion. Because the lesion remained firm and intermittently draining despite systemic steroids and local wound care, the gastrointestinal service requested pelvic magnetic resonance imaging (MRI) on hospital day 7. Pelvic MRI revealed a rectocutaneous fistula originating from the distal rectum and extending through the left perianal soft tissues to the area corresponding to the cyst in the gluteal region (Figure 3). At that time, he was transitioned from intravenous methylprednisolone to an oral prednisone taper and discharged with close gastrointestinal and surgical follow-up.

**Figure 3.**
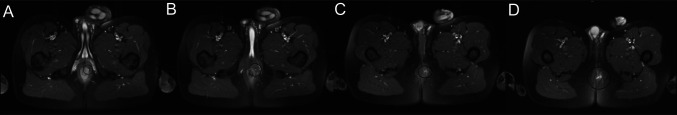
Serial pelvic magnetic resonance imaging with contrast images (A–D) demonstrating a rectocutaneous fistulous tract (circled) arising from the distal anorectal/anal canal region and extending leftward through the perineal soft tissues into the left gluteal region.

At the initial surgical consult 4 weeks later, he reported ongoing fluctuating tenderness and intermittent drainage without systemic symptoms. Examination at that visit documented a left lateral external opening <1 cm in diameter with scant purulent drainage, without surrounding erythema, induration, or tenderness. Operative intervention was ultimately deferred, with a plan to complete the steroid taper and medically optimize his UC maintenance therapy. He subsequently initiated infliximab at 5 mg/kg (350 mg per infusion, administered with standard induction at weeks 0, 2, and 6 followed by every-8-week maintenance) and azathioprine 75 mg daily. Repeat surgical evaluation 8 weeks later noted a single small external opening with minimal discharge, interval healing of 2 previously draining openings, and no induration or tenderness. The fistula was described as healing, and no operative intervention was acutely recommended with continued surveillance planned.

## DISCUSSION

Rectocutaneous fistulas are rare in UC because UC is classically characterized by mucosal, rather than transmural inflammation. The presence of a rectocutaneous fistula in a patient with UC should therefore trigger careful diagnostic reassessment for Crohn's disease or indeterminate colitis. Cohort data suggest that such a diagnostic change is at least as common as fistula formation in confirmed UC: in 1 large series, approximately 14% of patients with an initial inflammatory bowel disease (IBD) diagnosis had a change in classification over time, and 1.7% of those referred with UC were ultimately rediagnosed with Crohn's disease.^13^ In our patient, the working diagnosis remained severe left-sided UC, as supported by the continuous Mayo 3 proctosigmoiditis, a normal proximal colon and terminal ileum, supportive histology, absence of skip lesions or ileitis, and no prior history of recurrent PAD. We therefore considered Crohn's disease or indeterminate colitis a longitudinal diagnostic consideration rather than the most accurate classification at this time.

The likely origin of the fistula was also considered. A cryptoglandular fistula remained a plausible alternative because the patient initially described a localized gluteal boil that would intermittently enlarge, fill up, and drain. However, several features favored an IBD-associated fistula. First, the lesion developed after an episode of severe rectal pain, and persisted until hospitalization for a UC flare. Second, pelvic MRI revealed a tract originating from the distal rectal/anorectal region and extending to the same left gluteal site previously thought to represent a cyst. Finally, the subsequent improvement in the external openings after escalation of UC-directed therapy with infliximab and azathioprine, without operative intervention, further supports an inflammation-associated process. Therefore, we characterize the tract as most consistent with UC-associated fistulizing disease arising during severe active proctosigmoiditis, while acknowledging cryptoglandular disease and future diagnostic reclassification as important longitudinal considerations.

Management of rectocutaneous fistulas in IBD centers on sepsis control, accurate anatomic delineation, and medical optimization of underlying bowel inflammation.^14^ For complex perianal Crohn's disease, contemporary guidelines and reviews support a combined medical-surgical strategy in which setons may be used to drain the tract and preserve sphincter function, while biologic therapy—most commonly anti-tumor necrosis factor agents such as infliximab, often in combination with a thiopurine—promotes fistula healing and maintains remission.^15,16^ While data in UC is far more limited, there are reports to suggest that infliximab-based regimens can similarly induce closure of perianal fistulas.^17^ In our patient, the small external opening, lack of fluctuance or systemic sepsis, and early symptomatic improvement allowed the multidisciplinary team to defer immediate operative intervention and instead prioritize pharmacologic optimization of his UC, leading to early fistula healing.

This case underscores several practical lessons. First, a chronic, draining, nonhealing perianal or gluteal lesion should prompt consideration of fistula, regardless of IBD subtype. While Crohn's disease remains the dominant association, UC does not exclude fistulizing disease.^18^ Second, it is possible for the physical examination, imaging, and endoscopy to miss complex or extrasphincteric tracts. Our patient's colonoscopy, digital examination, and ultrasound failed to demonstrate a connection to the rectum. Dedicated pelvic MRI is more sensitive for mapping perianal fistulas, and should be obtained early when the clinical image is discordant or symptoms persist.^19^ Finally, reassessment is crucial when a benign diagnosis does not explain the full clinical course. In hindsight, the coexistence of severe proctosigmoiditis, history of extraintestinal ulceration/abscess formation, and the presence of a long-standing draining buttock lesion should have lowered the threshold for pelvic imaging earlier. In conclusion, this case illustrates that rectocutaneous fistulas, though rare, can complicate UC, and should remain in the differential diagnosis when a chronically draining buttock lesion persists with no clear etiology.

## DISCLOSURES

Author contributions: R. Sasse: Writing—review and editing, Writing—original draft, investigation, visualization, validation, conceptualization. N. Hassan, M. Hassan, V. Mateescu: Writing—review and editing, investigation, validation. TM Mahmoudi and E. Sadeddin: Writing—review and editing, validation. R. Sasse is the article guarantor.

Financial disclosure: None to report.

Informed consent was obtained for this case report.

## ABBREVIATIONS:

GI: gastrointestinal
IBD: inflammatory bowel disease
MRI: magnetic resonance imaging
PAD: perianal disease
UC: ulcerative colitis
